# Modeling and X-ray Analysis of Defect Nanoclusters Formation in B_4_C under Ion Irradiation

**DOI:** 10.3390/nano12152644

**Published:** 2022-07-31

**Authors:** Matlab N. Mirzayev, Alexander A. Donkov, Evgeni A. Popov, Ertugrul Demir, Sakin H. Jabarov, Levan S. Chkhartishvili, Samuel A. Adeojo, Aleksandr S. Doroshkevich, Alexey A. Sidorin, Asif G. Asadov, Thabsile T. Thabethe, Mayeen U. Khandaker, Sultan Alamri, Hamid Osman, Alex V. Trukhanov, Sergei V. Trukhanov

**Affiliations:** 1 Institute of Radiation Problems, Azerbaijan National Academy of Sciences, Baku AZ-1143, Azerbaijan; sh_jabarov@mail.ru; 2 Scientific-Research Institute Geotecnological Problems of Oil, Gas and Chemistry, Azerbaijan State Oil and Industry University, Baku AZ-1010, Azerbaijan; 3 Joint Institute for Nuclear Research, Dubna 141980, Russia; aa_donkov@mail.ru (A.A.D.); ep_popov@mail.ru (E.A.P.); as_doroshkevich@mail.ru (A.S.D.); aa_sidorin@mail.ru (A.A.S.); ag_asadov@mail.ru (A.G.A.); 4 Institute of Solid State Physics, Bulgarian Academy of Sciences, 1784 Sofia, Bulgaria; 5 Institute for Nuclear Research and Nuclear Energy, Bulgarian Academy of Sciences, 1784 Sofia, Bulgaria; 6 Physics Department, Yeditepe University, Istanbul 34755, Turkey; er_demir@mail.ru; 7 Georgian Technical University, Tbilisi, 77, Kostava Str., 0160, Georgia; ls_chkhartishvili@mail.ru; 8 Ferdinand Tavadze Metallurgy and Materials Science Institute, Tbilisi, 8b, E. Mindeli St., 0186, Georgia; 9 Department of Physics, University of Pretoria, Pretoria 0002, South Africa; sa_adeojo@mail.ru (S.A.A.); tt_thabethe@mail.ru (T.T.T.); 10 Donetsk Institute for Physics and Engineering Named after O.O. Galkin NAS of Ukraine, 03028 Kyiv, Ukraine; 11 Institute of Physics, Azerbaijan National Academy of Sciences, Baku AZ-1143, Azerbaijan; 12 Centre for Applied Physics and Radiation Technologies, School of Engineering and Technology, Sunway University, Bandar Sunway 47500, Malaysia; mayeenk@sunway.edu.my; 13 Department of General Educational Development, Faculty of Science and Information Technology, Daffodil International University, DIU Rd, Dhaka 1341, Bangladesh; 14 Radiological Sciences Department, College of Applied Medical Sciences, Taif University, Taif 21944, Saudi Arabia; S.Alamri@tu.edu.sa (S.A.); ha.osman@tu.edu.sa (H.O.); 15 Laboratory of Magnetic Films Physics, SSPA “Scientific and Practical Materials Research Centre of NAS of Belarus”, 19, P. Brovki str., 220072 Minsk, Belarus; truhanov86@mail.ru; 16 Smart Sensors Laboratory, Department of Electronic Materials Technology, National University of Science and Technology MISiS, 119049 Moscow, Russia

**Keywords:** defect formation, boron carbide, defect vacancies, TCDFT, PLT

## Abstract

In the presented work, B_4_C was irradiated with xenon swift heavy ions at the energy of 167 MeV. The irradiation of the substrate was done at room temperature to a fluence of 3.83 × 10^14^ ion/cm^2^. The samples were then analyzed with the X-ray diffraction technique to study the structural modification, as it can probe the region of penetration of xenon atoms due to the low atomic number of the two elements involved in the material under study. The nano-cluster formation under ion irradiation was observed. Positron lifetime (PLT) calculations of the secondary point defects forming nanoclusters and introduced into the B_4_C substrate by hydrogen and helium implantation were also carried out with the Multigrid instead of the K-spAce (MIKA) simulation package. The X-ray diffraction results confirmed that the sample was B_4_C and it had a rhombohedral crystal structure. The X-ray diffraction indicated an increase in the lattice parameter due to the Swift heavy ion (SHI) irradiation. In B_12_-CCC, the difference between τ with the saturation of H or He in the defect is nearly 20 ps. Under the same conditions with B_11_C-CBC, there is approximately twice the value for the same deviation.

## 1. Introduction

Boron-based materials, such as boron carbide (B_4_C), are widely used in a number of applications, such as nuclear technology, solar energy, electronics, engineering and refractory application because of their excellent properties [1,2,3,4]. These properties include high melting point, thermal stability, exceptional abrasion, high hardness, low density and high thermal neutron absorption cross-section [5,6,7,8]. B_4_C-based composites are also used as thermoelectric materials, which allows the transformation of thermal energy especially at high temperatures to electrical energy [9,10]. The exceptional properties of such materials, including their ability to resist change in variable temperatures, puts them in high demand. Several researchers have reported on the heat treatment and irradiation by gamma, electron, neutron [11,12,13] and swift ions irradiation of boron composites [14,15,16,17].

The reports have explained the mechanism for the formation of defects caused by irradiation, the migration on energy levels of defects and the recombination kinetics due to heat treatment [18,19,20,21]. It is well known that ion irradiation is one of the frequently used methods for obtaining clusters of various sizes [22]. However, extremely high current density is often required for cluster formation, so it is believed that the efficiency of nanocluster formation by ion irradiation is somewhat lower than by other methods [23]. Nanoclusters can be easily obtained using reactive ions [24,25,26]. The surface reaction between the irradiated ion and the target atom will lead to the formation of various nanoclusters, which are difficult to obtain by other methods, since they are based on reactions proceeding through nonequilibrium processes [27].

Studies focusing on B_4_C have generally explained the amorphization mechanism due to gamma irradiation, the change in the distance between atoms, displacement of space planes and the ratio of lattice parameters [28,29]. A few studies have investigated the microstructural changes of B_4_C influenced by swift heavy ion (SHI) irradiation and the formation of pinholes [5,30,31]. In this paper, we emphasized the properties of B_4_C as a potential candidate for fusion-type reactor material [32]. That is, we are launching a preparatory study on the possible participation of B_4_C material as one of the materials that can be used as the first wall blanket and diverter in International Thermonuclear Experimental Reactor (ITER) [33,34]. The aim of this paper is to determine the possible defects that will form in B_4_C when irradiated with ions and neutrons and to predict the evolution of those defects [35].

For example, the diverter of a thermonuclear reactor is ligated with a special coating including several components, from which sintering is obtained as a product of our desired material. Such materials can be a combination of WB in its various phases—W_2_B, W_5_B_2_, WB_2_, which should be sintered from nanopowder in a certain ratio of input materials—W and B_4_C. Within centimeters, the temperature gradient varies over a very wide range. Assuming that in situ-sintered materials, such as W_2_B, are used, it can be expected that in areas where the temperature is not sufficient, there will be zones containing the base materials, such as B_4_C. The products and participants in the fusion, such as the deuterium (D), tritium (T), helium (He), and n neutrons, continuously attack the walls and the diverter. After the walls and diverter coating of a thermonuclear reactor are attacked by high-energy neutrons and light ions, in addition to bulk defects, a secondary ionizing wave of heavy ions occurs, which is a consequence of the first ionizing agents. Therefore, one would then expect that heavy elements of the material under study, such as tungsten, would exhibit secondary ionization, modeled here by Xe^26+^ xenon ions. The latter will affect the adjacent areas of the material under study, consisting of B_4_C, and together with hydrogen, helium and neutrons, will participate in the creation of defects. This is the reason we study structural defects, such as volume vacancy nanoclusters filled with hydrogen and helium participants and products in the synthesis [36,37,38,39,40,41].

As already mentioned, there are two main processes in which defects occur. The one is from the irradiation with the particles that participate in the fusion synthesis, i.e., He, hydrogen (H), and neutrons (n). We consider the corresponding defects in this case in the context of positron annihilation with numerical methods. The other one is from the secondary wave of heavy atoms from the protective materials. For this situation, the produced damage is modeled by the use of Xe xenon as a hitting particle, as it is a close element to the assumed heavy particles from the firewall, and with an actual experiment with such SHI irradiation beam. Although initially Xe^26+^ xenon ions from the beam are far more energetic in comparison to the energies of the assumed heavy particles, such as W tungsten, which are in the range of 100 eV, near the end of the Xe particles path, they reach a comparable range of a few eV. These initial energetic Xe^26+^ xenon ions do not cause noticeable damage to the material under study since their energy drops quite quickly toward the end of the path. Accounting for these two processes is very important, and, to a certain extent, innovative, since it makes it possible to describe the formation of defect nanoclusters during irradiation in the most complete and adequate way.

The manuscript is organized as follows. First, we begin with the experimental setup in Section 2. Then, we discuss several results from the X-ray diffraction measurements in Section 3. Finally, Section 4 is devoted to the positron annihilation modeling results.

## 2. Experimental Section

In this study, B_4_C samples were prepared from a single boron carbide substrate. Boron carbide samples with a bulk density of 1.8 g/cm^3^, specific surface area of 2–4 m^2^/g, particle size of 3–7 μm and a purity of 99.9% (US Research Nanomaterials, Inc., Houston, TX, USA) were used in the experiments.

Before the irradiation, the substrates were cleaned using Struers LaboForce-50. The samples were then irradiated with 167 MeV 132Xe SHIs to a fluence of 3.83 × 10^14^ ion/cm^2^ at room temperature (RT). The irradiation was done at IC100 cyclotron in Flerov Laboratory for Nuclear Reactions (FLNR), Joint Institute for Nuclear Research (JINR) in Dubna, Russia. The temperature of the samples during irradiation did not exceed 50 °C. 

Samples prepared for irradiation are pasted on a Faraday cylinder. A Faraday cyl-inder is used to measure the temperature of the samples during irradiation. The Faraday cylinder is connected to a thermocouple. The temperature of the sample is determined by the current amperage and resistance in the cylinder. The temperature of the samples during irradiation did not exceed 50 °C.

The samples were analyzed using X-ray diffraction (XRD) (DKSH Technology Sdn. Bhd., Selangor, Malaysia), specifically, the wind angle X-ray scattering (WAXS) before and after irradiation to monitor the microstructural changes on B_4_C. A Cu-K_α_ radiation source with a wavelength of 1.5406 Å, operating voltage and current of 40 kV and 40 mA, respectively. The XRD pattern was analyzed with the Rietveld method using the Fullprof program [42]. The XRD is able to probe through the entire depth of our B_4_C sample, thus the XRD data graph shows signals from different zones in the sample: undamaged, as well as damaged and amorphized zones.

The radiation time is 556 h. The irradiation process was performed in monotonic mode.

## 3. Swift Heavy Ion Irradiation: X-ray Diffraction

**Figure 1:**
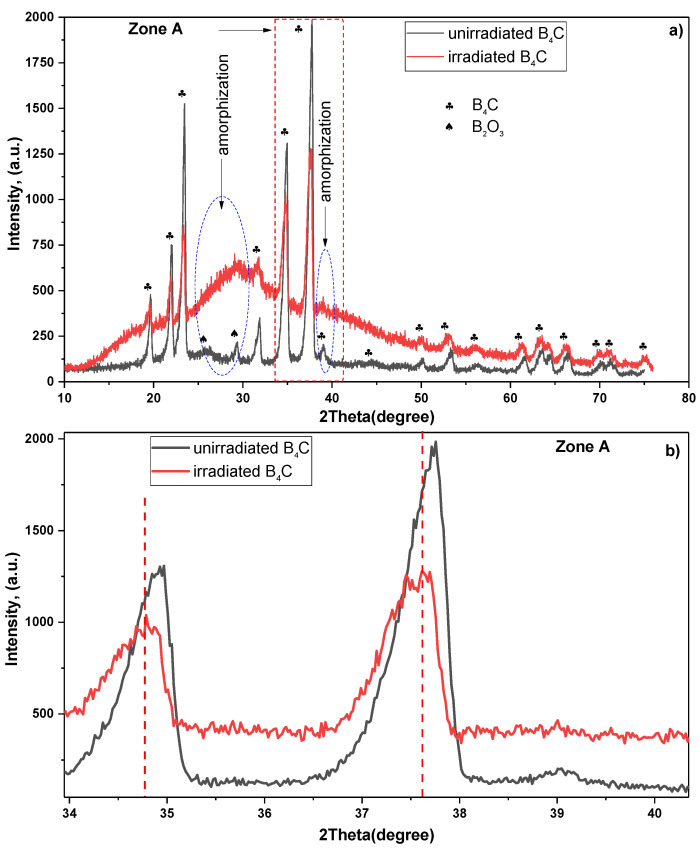
X-ray diffraction. On the top, (**a**) is the X-ray diffraction of unirradiated (black line) and irradiated boron carbide (red line), on the bottom, (**b**) is the enlarged part labeled as Zone A. The XRD is able to probe through the entire depth of our B_4_C sample; thus, the XRD data graph shows signals from different zones in the sample: undamaged, as well as the damaged and amorphized zones.

After irradiation of the material with heavy Xe^26+^ xenon ions, we used XRD experimental methodology to analyze the structural changes in the sample. The whole range of penetration of Xe xenon atoms is seen well enough by XRD due to the low atomic number of the two elements involved in the material we are studying. Figure 1 shows a shift of X-ray spectra in a B_4_C unirradiated and irradiated sample with 167 MeV energy the ^132^Xe^26+^ SHIs.

The XRD pattern for the unirradiated sample indicated several peaks of B_4_C, all assigned to a rhombohedral crystal structure. The rhombohedral crystal structure had a space group of $R\overset{-}{3}m$. The lattice parameters for the unirradiated samples were: a_XRD_ = 5.62922(4) Å and c_XRD_ = 12.13944(6) Å in hexagonal setting, which correlate with the theoretical values and previous results [43,44]. In addition, there are B-O covalent bonds on the surface of the unirradiated boron carbide sample, which are characterized by O oxygen atoms captured by active boron centers [45,46]. The capture of O oxygen atoms can be shown by the following mechanism:

B + O_2_ → [BO]• + O• O• + B → [BO]• [BO]• + O_2_ → [BO_2_]• + O• (1)

After irradiating the B_4_C samples with Xe^26+^ ions at a fluence of 3.83 × 10^14^ ion/cm^2^, the crystal structure of the samples was observed to change. The lattice parameters of the irradiated samples were obtained as a_XRD_ = 5.65747(4) Å and c_XRD_ = 12.19866(6) Å in hexagonal setting. The change in the lattice parameter when compared to the unirradiated samples indicates the partial lattice damage introduce by the SHI irradiation. That is, the irradiated lead to peak broadening and the disappearance of the peaks around the 2-theta position of 26° and 29° (Figure 1a). The disappearance of the peaks around 26° and 29° is due to amorphization. The disappearance of the intensity of the peaks confirms the mechanism of the process. The 2-theta position of 26° and 29° the disappearing peaks are weakly interacting B-O chemical bonds that are degraded under the influence of SHI. A slight peak shift toward the lower theta value along the x-axis was also observed. The peak broadening observed can be attributed to the partial amorphization of the B_4_C [47,48].

The change in the peak position can be due to the lattice disorder (lattice expansion) in the crystal structure and the stress introduced by the SHI irradiation (Figure 1b—Zone A) [49,50,51]. According to L. Desgranges et al. [52], displacement of X-ray diffraction spectra was observed in a sample of B_4_C boron carbide irradiated with thermal neutrons. Peak displacement and decrease in intensity are characterized by the deviation of atoms from the coordinates of the crystal lattice, i.e., formation of defect cascade and the degradation or amorphization of the crystal structure under the influence of high-energy ion streams [53,54]. The crystallite size, determined with help of the Scherrer equation, is shown in Table 1 for the unirradiated and irradiated B_4_C boron carbide sample under 167 MeV energy ^132^Xe^26+^ SHIs at 3.83 × 10^14^ ion/cm^2^. The irregular behaviour of the FWHM and crystallite size of the two peaks at the 2 × theta = 31.77(2)° and 61.51(2)° compared to others as a result of the effect of SHI in the BC compound can be explained by the construction of some chemical bonds in that structure.

From Table 1, shift of peaks, disappearing of peaks, full width half-maximum expansion and decrease of crystallite size in irradiated B_4_C boron carbide are distributed in the formation of small nanocrystal centers because of the high kinetic energy transmitted by the SHI of the crystal. The value of crystallite size increases only at the boron carbide peak, which corresponds to the 2-theta position of 61.3°. It is believed that a small amount of new B-C icosahedron is formed as a result of the SHI effect. Reduced grain size with the increasing fluence should improve the sintering properties of the materials that would be used in natural sintering in thermonuclear reactors. The additional fragmentation introduced by the irradiating ions should further ease this sintering process.

The XRD pattern indicated that there was partial amorphization in the B_4_C crystal structure with parts of the B_4_C structure still intact. This indicated that the B_4_C material has the ability to retain its crystal structure during swift heavy ion irradiation and the ionization process. This ability to resist severer damage is useful when considering the ability of candidates to protect plasma from contamination after secondary ionization. Also, from XRD is seen an increase in the number of volumetric defects after irradiation.

## 4. Model Calculations of PLT

**Figure 2:**
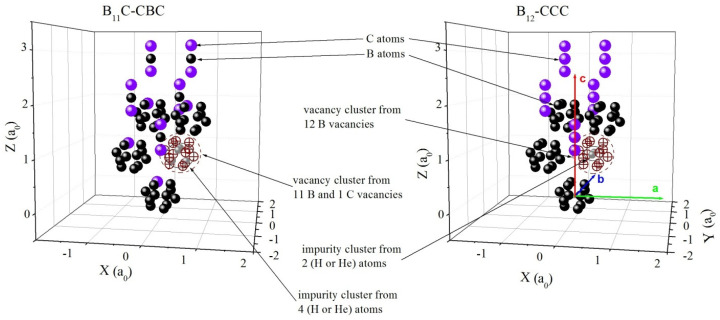
Visualization of super cell of B_4_C and positions of point defects in vacancy cluster from 12 vacancies impurity with H or He atoms. b = 2a_0_ × sin(α/2), α = 65.981° a rhombohedral setting. There are presented two cases: fist polarized B_11_C-CBC and second B_12_-CCC.

In this part of the manuscript structural defects were considered, from volume vacancy nanoclusters and which are filled with hydrogen and helium. With the help of software package MIKA [55,56], a series of positron lifetime calculations for the B_4_C medium were done employing the local density approximation (LDA) and general gradient approximation (GGA) algorithms. A super cell from 75 atoms with 60 B atoms and 15 C atoms was constructed for the calculations. The lattice parameters were kept fixed to the results from XRD in the orthogonal coordinate system. In order to keep the original symmetry and to align with the values from XRD, b = a_XRD_, a = 2bcos30° and c = c_XRD_ were taken. This can be seen in Figure 2.

In order to clarify the methodology we use in the model calculations, we will make a short deviation from the theory built into the MIKA program. This is the model of the two-component extension of the density functional theory (TCEDFT) [57]. In a concise form, it is necessary to emphasize that part of the general functional that corresponds to the exchange correlation interaction. The exchange correlation energy has been expressed with density of the particles—*n*(*r*) [58,59]:

(2) $$E_{xc}\left[n\left(r\right)\right]=\int n\left(r\right)\varepsilon_{xc}\left[n\left(r\right)\right]dr,E_{xc}\left[n\left(r\right)\right]=\int n\left(r\right)\varepsilon_{xc}\left[n\left(r\right)\right]dr,$$

where $\varepsilon_{xc}\left[n\left(r\right)\right]$ represents the one-particle functional of the exchange-correlation energy. The exchange correlation energy falls below the general formulation of the effective potential of Kohn and Sham in their one-particle equation [60]:

(3) $$\left\{-\frac{1}{2}\nabla^{2}+V_{eff}\left[n\left(r\right),r\right]\right\}\varphi_{i}\left(r\right)=\varepsilon_{i}\varphi_{i}\left(r\right),$$

Equation (3) represents the single-particle Schrödinger equation with a form identical to that of the Schrödinger equation for non-interacting particles in an external potential. The DFT formalism [58,59], proposed by Hohenberg, Kohn and Sham, is suitable for the interaction between electrons and positrons. The positron wave function *φ*_+_(*r*) is calculated as follows [57]:

(4) $$n_{+\left(r\right)=\underset{i}{\sum }\varphi_{+{\left(r\right)}^{2},}}$$

The positron annihilation rate λ in an inhomogeneous electron gas is proportional to the overlap of electron *n*−(*r*) and positron *n*_+_(*r*) densities in the LDA. The GGA method in the enhancement factor uses adjustable parameter α that is connected with LDA by this equation: *g_GGA_* = 1 + (*g_LDA_*-1)*e^-αε^*, where g is the enhancement factor and ε is the electron density. The annihilation rate *λ* is the reciprocal value of the positron lifetime, in an inhomogeneous electron gas:

(5) $$\lambda =\pi r_{0}^{2}c\int drn_{+\left(r\right)n_{-\left(r\right)}\gamma =\pi r_{0}^{2}c\sum_{i}\int dr}$$

where *r*_0_ is the classical electron radius, *c* is the speed of light and *γ* is the enhancement factor of the electron density at the positron. In this case, the iterative procedure of the program works with precision for the convergence with twelve decimal places. Due to the low atomic number and loose structure, in combination with the non-metallic properties of the material, a low electron density in the basic medium is assumed. For this reason, there were many difficulties with the calculations. The *τ* positron lifetime in a growing vacancy cluster was calculated and the defect behavior when B_4_C was implanted with H and He atoms was studied. Two configurations of this material—B_11_C_p_-CBC and B_12_-CCC, where C_p_ means C is in polar position in a B-icosahedron, were explored [35]. Taking into account the data from the XRD, the lattice parameters in particular, it can be assumed that the material is more likely to be in B_11_C-CBC form. The results are presented in Table 2.

The results from the *τ* calculation for a perfect lattice on B_4_C are in satisfactory agreement with *τ_2_* that was reported by Liu [47], where the *τ*_2_ value cannot be attributed to cavities and grain boundaries, which we interpret as not to be due to those cavities and grain boundaries. This value can be considered as coming from annihilation of the positron in areas outside the icosahedrons, where a sufficiently low electron density is expected. After irradiation with ions (H/He) or neutrons, defects of vacancy cluster type are expected to be produced. Here, we numerically model such vacancy clusters, and the values in Table 2 give the dependence of τ on the increase in vacancy volumes and implanted H/He atoms in them. The picture shown in Figure 2 represents the largest volume of the defect which was be created in our super cell of B_4_C, with the vacancy cluster being created by successively removing the nearest boron atoms to the defect position.

The cases of implanted H and He atoms in the internodes were also considered. The lower *τ* value of the B_11_C-CBC combination compared to B_12_-CCC confirms that the more physically optimized version of B_4_C is B_11_C-CBC [35]. Apart from Liu’s work [44], positron spectroscopy was performed in several other publications [53,61,62]. It is significant that the Doppler broadening of the spectrum is mainly considered in them, but the *τ* results are somehow neglected. In our work, we have tried to make up for this omission.

There is some controversy in the values of the single lifetime spectrum in the present work and works [47,62]. The reasons for this difference remain to be elucidated in further studies. In [62] a single lifetime spectrum of *τ* = 166 ps was observed, which is significantly lower than the values of *τ* for bulk from Table 2, which are between 340 and 470 ps. It should be noted that a similar lifetime component, 166.2 ps, dominated the lifetime spectrum in Ref. [47] as well. As a rule, there are only minor mutual differences between the current calculated lifetimes for bulk samples, as well as for 1 V and 11/12 V ones. The calculated lifetime for some defects or intermediate configurations turns out to be even lower than the corresponding values of the bulk samples. An interesting question is whether each of the types of defects considered here is capable of capturing positrons. The key parameter of such capture is the binding energy of the positron with a particular type of defect. However, this issue was not considered in this manuscript, so the values of the positron binding energy are not given here. In addition, the potential role of atomic relaxations is also not discussed in this manuscript.

**Figure 3:**
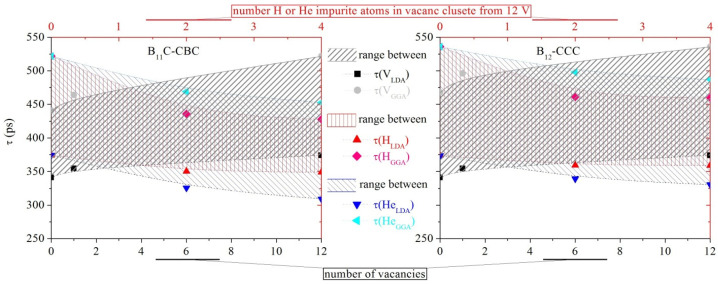
Functional dependencies between *τ* and number of vacancies or H and He atoms in vacancy cluster from 12 vacancies. These ranged between the *τ* functions of LDA and GGA methods.

The number of vacancies to the *τ* and the number of impurities to τ were plotted and presented in Figure 3. A slight deviation of the *τ* was observed regardless of the type of atoms removed or added to the B_12_-CCC. Accordingly, the difference between *τ* with the saturation of H or He defect is nearly 20 ps. If a comparison is made under the same conditions with B_11_C-CBC, approximately twice the value for the same deviation will be seen. The range between LDA and GGA in B_12_-CCCis wider than in B_11_C-CBC. This should be interpreted as a better proximity to the physical optimization of the modification B_11_C-CBC. Experimental results should be in the presented ranges between LDA and GGA results. Overall, a relatively small range of variation means that the type of defect in the B_4_C material will give negligible change in the electron density.

At the same time, we can conclude that in areas with a small temperature gradient, sintered materials with the B_4_C component may be suitable for removing helium or hydrogen from the reactor walls which are undesirable after synthesis. A substantial reason for this conclusion is due to the low electron density and large free volumes of the B_4_C material.

In this work, the corresponding limited number of atoms in the supercell, namely 15C and 60B have been used. We used the above-mentioned articles and added the results for the (B11Cp)CBC polytype, and also considered the mentioned vacancies and He atoms in interstitial positions with the results presented in the top few rows of Table 2. As already mentioned, there are two main processes in which defects occur. One process arises from the irradiation with the particles involved in thermonuclear fusion, i.e. He, H and the neutron. We consider the corresponding defects in this case in the context of positron annihilation by numerical methods. Another process arises from a secondary wave of heavy atoms from the protective materials. For this situation, the damage produced was modeled using Xe^26+^ ions as the incident particle, since it is a close element to the assumed heavy particles from the firewall, and with a real experiment with such an SHI irradiation beam. In addition, we intend to conduct experimental studies on SEM and study mechanical properties.

## 5. Conclusions

An analysis of B_4_C was carried out with the aim of identifying its potential application at a typical international thermonuclear experimental reactor. Specifically, the B_4_C material was irradiated with ^132^Xe^26+^ SHIs to a fluence of 3.83 × 10^14^ ion/cm^2^ at room temperature. The samples before and after irradiation were characterized with XRD to study the structural changes and defect nanocluster formation. The positron lifetime (*τ*) calculations were carried out to predict the evolution of defect behavior when B_4_C was implanted with hydrogen and helium atoms. The XRD result shows that the lattice parameters increase because the irradiated heavy atoms fill the volume of cells of B_4_C substrate. On the other hand, a partially amorphized B_4_C structure was observed after irradiation. After irradiation, fragmentation of the grains was observed, which leads us to believe that in the natural sintering of base materials, this will be a beneficial effect. The change in the electron density with increasing volume defects is relatively small, which implies good radiation resistance. It can be said that the material is suitable for natural sintering of basic components in a thermonuclear reactor.

## Figures and Tables

**Table 1:** Crystallite size of the unirradiated and irradiated B_4_C boron carbide sample under 167 MeV energy ^132^Xe^26+^ SHIs at 3.83 × 10^14^ ion/cm^2^.

*K*	*λ(Å)*	*Peak Position (2θ) of Unirradiated Boron Carbide Sample*	*Peak Position (2θ) of Irradiated Boron Carbide Sample*	*FWHM of Unirradiated Boron Carbide Sample*	*FWHM of Irradiated Boron Carbide Sample*	*Crystallite Size of Unirradiated Boron Carbide Sample, L (nm)*	*Crystallite Size of Irradiated Boron Carbide Sample, L (nm)*
*0.94*	*1.54178*	*19.61(2)*	*←19.51(2)*	*0.31(3)*	*0.42(3)↑*	*26.78(2)*	*19.84(2)↓*
		*21.97(2)*	*←21.86(2)*	*0.33(3)*	*0.37(3)↑*	*25.45(2)*	*22.56(2)↓*
		*23.40(2)*	*←23.32(2)*	*0.34(3)*	*0.39(3)↑*	*24.82(2)*	*21.56(2)↓*
		*29.29(2)*	*-*	*0.39(3)*	*-*	*21.86(2)*	*-*
		*31.77(2)*	*←31.49(2)*	*0.51(3)*	*1.98(3)↑*	*17.02(2)*	*4.36(2)↓*
		*34.82(2)*	*←34.69(2)*	*0.49(3)*	*0.52(3)↑*	*17.92(2)*	*16.76(2)↓*
		*37.63(2)*	*←37.53(2)*	*0.51(3)*	*0.56(3)↑*	*17.32(2)*	*15.65(2)↓*
		*38.00(2)*	*-*	*0.59(3)*	*-*	*14.89(2)*	*-*
		*50.05(2)*	*-*	*0.76(3)*	*-*	*12.12(2)*	*-*
		*53.25(2)*	*←53.02(2)*	*0.79(3)*	*1.01(3)↑*	*11.74(2)*	*9.23(2)↓*
		*61.51(2)*	*←61.32(2)*	*0.81(3)*	*0.63(3)↓*	*11.94(2)*	*15.32(2)↑*
		*63.48(2)*	*←63.33(2)*	*1.03(3)*	*1.54(3)↑*	*9.52(2)*	*6.34(2)↓*
		*66.38(2)*	*←66.21(2)*	*1.04(3)*	*1.06(3)↑*	*9.51(2)*	*9.35(2)↓*

**Table 2:** The calculated values for τ in a growing nanocluster of point defects and H and He implantation.

B_11_C-CBC			B_12_-CCC		
Novacancies and Impurity Atoms	*LDA τ* (ps)	*GGA τ* (ps)	Novacancies and Impurity Atoms	*LDA τ* (ps)	*GGA τ* (ps)
bulk	341	441	bulk	348	468
3H interstitials	286	334	3H interstitials	312	386
3He interstitials	258	355	3He interstitials	280	394
1V_B_	354	464	1V_B_	363	496
1V_C_	354	464	1V_C_	363	496
1V_B_ in center of the chain	354	464	1V_C_ in center of the chain	363	496
1V_C_ + 11V_B_	374	522	12 V_B_	374	536
1V_C_ + 11V_B_ 1H nearly to C atom	373	516	12 V_B_ 1H nearly to C atom	373	580
1V_C_ + 11V_B_with 2H in center cluster	350	436	12 V_B_ with 2H in center cluster	359	461
1V_C_ + 11V_B_ 2H nearly to C	372.2	515	12 V_B_ 2H nearly to C	372.7	532
1V_C_ + 11V_B_with 4H in center cluster	349	428	12 V_B_ with 4H in center cluster	359	460
1V_C_ + 11V_B_with 2He in center cluster	326	469	12 V_B_ with 2 He in center cluster	339	498
1V_C_ + 11V_B_with 4He in center cluster	309	453	12 V_B_ with 4 He in center cluster	330	487

## Data Availability

Not applicable.
